# Production of *Trans*-Cinnamic Acid by Immobilization of the *Bambusa oldhamii* BoPAL1 and BoPAL2 Phenylalanine Ammonia-Lyases on Electrospun Nanofibers

**DOI:** 10.3390/ijms222011184

**Published:** 2021-10-17

**Authors:** Pei-Yu Hong, Yi-Hao Huang, GiGi Chin Wen Lim, Yen-Po Chen, Che-Jen Hsiao, Li-Hsien Chen, Jhih-Ying Ciou, Lu-Sheng Hsieh

**Affiliations:** 1 Department of Food Science, Tunghai University, No. 1727, Sec. 4, Taiwan Boulevard, Xitun District, Taichung 40704, Taiwan; g08621018@thu.edu.tw (P.-Y.H.); g09621001@thu.edu.tw (Y.-H.H.); gg0680@gmail.com (G.C.W.L.); heinzchen@thu.edu.tw (L.-H.C.); jyciou@thu.edu.tw (J.-Y.C.); 2 Department of Animal Science, The iEGG and Animal Biotechnology Center, National Chung Hsing University, No. 145, Xingda Road, South District, Taichung 40227, Taiwan; chenyp@dragon.nchu.edu.tw; 3 Department of Ecology and Conservation Biology, Texas A&M University, 2126 TAMU College Station, TX 77843, USA; hsiaob@tamu.edu

**Keywords:** central composite design (CCD)-response surface methodology (RSM), dextran polyaldehyde, electrospun nanofiber, phenylalanine ammonia-lyase

## Abstract

Phenylalanine ammonia-lyase (PAL) catalyzes the nonoxidative deamination of phenylalanine to yield *trans*-cinnamic acid and ammonia. Recombinant *Bambusa oldhamii* BoPAL1/2 proteins were immobilized onto electrospun nanofibers by dextran polyaldehyde as a cross-linking agent. A central composite design (CCD)-response surface methodology (RSM) was utilized to optimize the electrospinning parameters. *Escherichia coli* expressed eBoPAL2 exhibited the highest catalytic efficiency among four enzymes. The optimum conditions for fabricating nanofibers were determined as follows: flow rate of 0.10 mL/h, voltage of 13.8 kV, and distance of 13 cm. The response surface models were used to obtain the smaller the fiber diameters as well as the highest PAL activity in the enzyme immobilization. Compared with free BoPALs, immobilized BoPALs can be reused for at least 6 consecutive cycles. The remained activity of the immobilized BoPAL proteins after storage at 4 °C for 30 days were between 75 and 83%. In addition, the tolerance against denaturants of the immobilized BoPAL proteins were significantly enhanced. As a result, the dextran polyaldehyde natural cross-linking agent can effectively replace traditional chemical cross-linking agents for the immobilization of the BoPAL enzymes. The PAL/nylon 6/polyvinyl alcohol (PVA)/chitosan (CS) nanofibers made are extremely stable and are practical for industrial applications in the future.

## 1. Introduction

**Figure 1:**
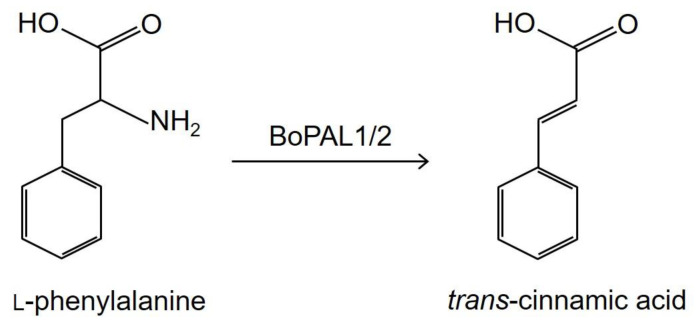
BoPAL1/2 catalyzed the deamination reaction to convert L-phenylalanine to *trans*-cinnamic acid.

Phenylalanine ammonia-lyase (PAL, 4.3.1.24) catalyzes the deamination of L-phenylalanine to yield trans-cinnamic acid and ammonia, is one of the crucial steps in the general phenylpropanoid pathway (Figure 1) [1,2]. PAL plays a vital role in supplying secondary metabolic products in plants such as flavonoids, lignins, caffeic acid, ferulic acid and so on [3,4]. Trans-cinnamic acid is well known to exhibit various health-promoting properties including anti-aging, anti-diabetic, anti-inflammatory, anti-microbial and anti-cancer activities [5,6,7,8]. The potential of trans-cinnamic acid as therapeutic agent for other conditions including Alzheimer’s disease, viral infections and tuberculosis [9]. In addition, recent studies found that trans-cinnamic acid has the potential effect to alleviate obesity [10,11]. Cinnamic acid derivatives are widely used in cosmetic industries such as perfumes, UV protecting agents [12] as well as possess anti-cancer, antioxidant and anti-microbial properties [13,14,15].

Enzymes are generally recognized as highly specific, versatile and environmental friendly catalysts which normally manipulate under gentle reaction conditions compared with chemical reactions [16]. However, free-form enzymes used in most of the processes are more susceptible to extreme temperature, pH and the presence of chaotropic agents [17]. For commercial viability, these biocatalysts display low operational stability and are difficult to recycle, leading to high costs. To solve the aforesaid limitation of free-form enzymes, enzyme immobilization now provides useful tools for improving the performance of enzymes for industrial applications [18,19]. In recent decades, functional nanomaterials have attracted attentions due to their unique properties and interesting applications. For examples, nanofibers, nanoparticles, and nanocomposites have been used as enzyme carriers [20] which able to provide large specific areas for a productive immobilization because of their extremely high surface area-to-volume ratios [21]. Electrospinning technique is an emerging nanotechnology in recent years which is a method that use an electrostatic force to process polymer solution into nanofibers toward a collector [22]. A high electric field is applied to the droplet of a fluid in which solution may come out from the tip, acting as one of the electrodes. Eventually the droplet is deformed from the tip of the cone, accelerating toward the adverse electrode and leading to the formation of continuous nanofibers [23,24,25]. Previously, several studies had pointed out the suitability of nanofiber as carrier in increasing the tolerance to extreme pH and temperature [26], promoting the reusability [27], prolonging the storage stability as well as enhancing the activities of the immobilized enzymes [28].

Recombinant BoPAL1 and BoPAL2 proteins were well-established and heterologously expressed in Escherichia coli and Pichia pastoris with high protein expression levels and purified to nearly homogeneity [29,30,31]. PAL immobilization was never been studied on electrospun nanofiber. To obtain the basic information, polyvinyl alcohol (PVA) and nylon 6 were wildly used in enzyme immobilization and chosen as supporting material of BoPAL enzymes due to its high biocompatibility as well as excellent structural stability in aqueous solutions [32].

## 2. Results

In this study, we made PVA/nylon 6 fibers in the presence of chitosan (CS) which provides amino group to fibers [32]. Combination of the electrospinning parameters (i.e., flow rate, applied voltage, and distance between spinneret and collector) were optimized using central composite design-response surface methodology (CCD-RSM). The aforementioned parameters within the context of RSM that produced nanofibers with the highest enzymatic activity as well as to study the interrelationship between PAL enzymatic activity and nanofiber diameter. The prepared immobilized PAL was characterized by Fourier transform infrared spectroscopy (FT-IR). The morphological characterization was monitored using thermal field emission scanning electron microscope (FE-SEM). The goal of this study was to compare PAL enzyme activity, optimum pH, optimum temperature, kinetic parameters (*k*_cat_ and *K*_m_), storage stability, and chemical stability in the immobilized and free BoPAL proteins.

### 2.1. Purification of Recombinant Proteins by Affinity Chromatography

**Figure 2:**
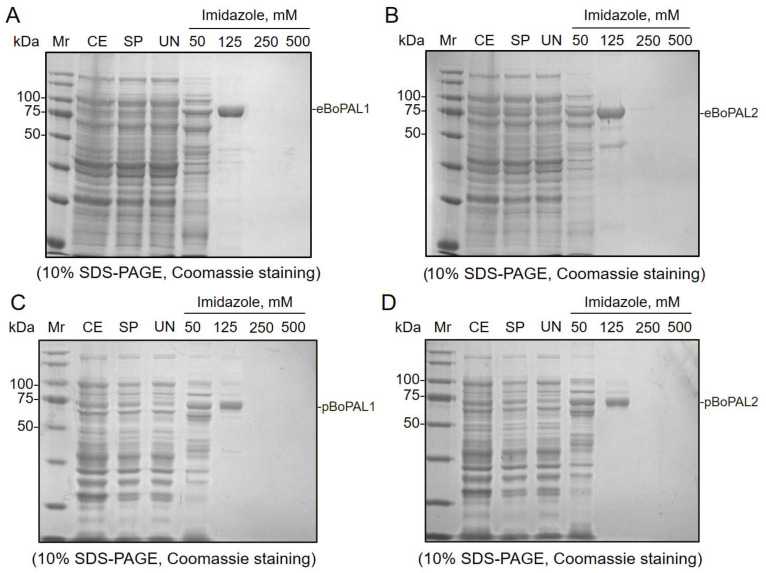
Purification of recombinant BoPAL proteins in *Escherichia coli* and *Pichia pastoris*. (**A**), eBoPAL1 was expressed and purified in *E. coli*; (**B**), eBoPAL2 was expressed and purified in *E. coli*; (**C**), pBoPAL1 was expressed and purified in *P. pastoris*; (**D**), pBoPAL2 was expressed and purified in *P. pastoris*. Recombinant BoPAL proteins were purified using Ni NTA resin and analyzed using 10% SDS-PAGE then stained with Coomassie Brilliant Blue-G250. Mr, Bio-Rad precision plus dual color standards; CE, crude extract; SP, soluble protein; UN, unbound protein. Ni NTA bound proteins were fractionated and eluted by various concentration of imidazole buffers.

The recombinant BoPAL proteins (Table S1) were purified on a Ni NTA column and eluted using different concentrations of imidazole buffers (Figure 2). *E. coli* expressed eBoPAL1 (Figure 2A), eBoPAL2 (Figure 2B) and *P. pastoris* expressed pBoPAL1 (Figure 2C), pBoPAL2 (Figure 2D) were eluted to nearly homogeneity at 125 mM imidazole buffer. On SDS-gels, major monomeric PAL bands were with a molecular mass of about 75 kDa (Figure 2A–D). The purification results were corresponded with previous reports [29,30,31,33]. The eBoPAL2 exhibited the best catalytic properties and used to measure the optimum conditions of protein immobilization. The other three forms of BoPALs were followed the reaction conditions obtained from eBoPAL2.

### 2.2. Optimum Crosslinking Conditions

**Figure 3:**
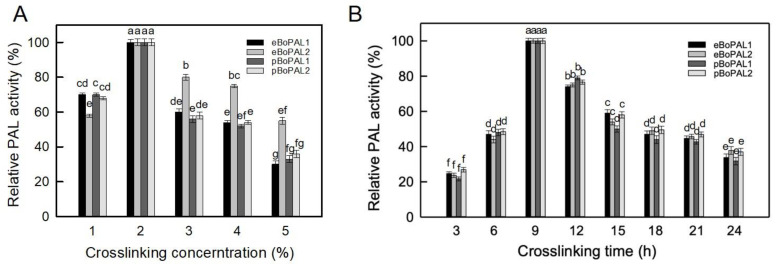
Optimum crosslinking conditions. (**A**), recombinant proteins were incubated with a range of concentration of dextran polyaldehyde varied from 1 to 5% for 3 h followed by PAL activity assay. (**B**), recombinant proteins were incubated with 2% dextran polyaldehyde for 3 to 24 h followed by PAL activity assay. All experiments were performed triplicate and expressed as average ± S.D. (error bars). Different letters were referred to significantly different (*p* < 0.05).

In this experiment, dextran polyaldehyde was prepared and utilized as a harmless and biodegradable natural macromolecular crosslinking agent to immobilize recombinant BoPAL proteins on PVA/nylon 6/CS nanofiber membrane [34]. Sodium periodate was used to oxidize the hydroxyl groups at the C-2 and C-3 positions on the dextran to produce di-aldehyde groups, which were then cross-linked with the amine groups on the enzyme and chitosan [34]. Thus, the cross-linking parameters (crosslinker concentration and cross-linking time) are the key considerations of protein immobilization. In Figure 3A, showed that the maximum PAL activities were detected consistently at a cross-linking concentration of 2%. When the crosslinking concentrations were further increased, the structure of the enzyme would be distorted and the activity would be lost. Similar phenomenon was reported by Sojitra et al., 2017 [34]. In Figure 3B, maximum PAL activities were remained on the immobilized BoPALs after 9 h. When the cross-linking time is further increased, the activity of PAL would gradually decrease presumably due to excessive cross-linking [35]. Taken together, 2% crosslinker and 9 h crosslinking time were suitable for immobilization BoPALs onto nanofiber membranes.

### 2.3. Central Composite Design (CCD)-Response Surface Methodology (RSM)

A central composite design (CCD) was employed to design the experiments. Actual and coded values of the variables were shown in Table S2. According to CCD design, three factors consists of 20 experiments included 8 corner points, 6 axial points and 6 replicates of the center points were applied to estimate the pure error sum of squares. The results at each point based on CCD and corresponded responses for each trail were shown in Table S3. Quadratic regression model was obtained to predict the PAL activity (2) and fiber diameter (3) of the produced nanofibers, respectively.

Y_1_ = 2067 − 11461 X_1_ − 134.1 X_2_ − 61.4 X_3_ + 22481 X_1_^2^ + 4.13 X_2_^2^ + 0.07 X_3_^2^ + 144 X_1_X_2_ + 458 X_1_X_3_ + 0.58 X_2_X_3_ (1)

Y_2_ = −671 + 5085 X_1_ + 86.9 X_2_ − 9.1 X_3_ − 4590 X_1_^2^ − 2.97X_2_^2^ + 1.24 X_3_^2^ − 122 X_1_X_2_ − 217 X_1_X_3_ + 0.42 X_2_X_3_ (2)

**Figure 4:**
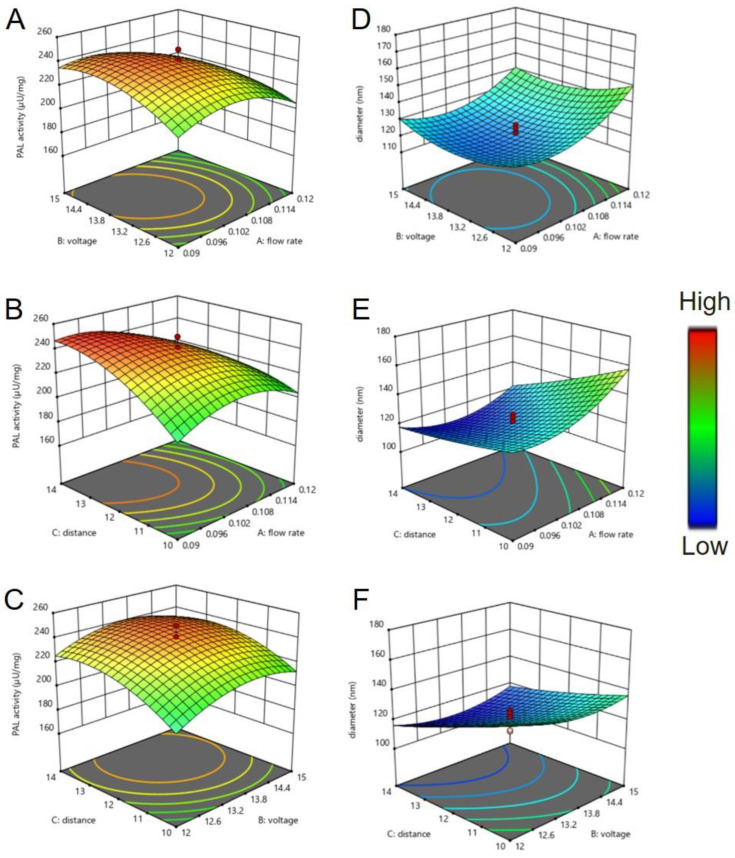
Determine of optimum electrospun nanofibers by response surface methodology. (**A**), effects of flow rates and voltages on PAL activity. (**B**), effects of flow rates and distances on PAL activity. (**C**), effects of voltages and distances on PAL activity. (**D**), effects of flow rates and voltages on nanofiber diameter. (**E**), effects of flow rates and distances on nanofiber diameter. (**F**), effects of voltages and on nanofiber diameter. Red and blue colors indicate high and low values, respectively.

According to Equation (1), theoretical highest of PAL activity was determined to be 253 (μU/mg NF) under the optimal conditions, where the flow rate, applied voltage, and distance between spinneret and collector were 0.10 mL/h, 13.8 kV and 13 cm, respectively. The relations between the input control factors (the electrospinning parameters) and the response variables (PAL activity and diameter of nanofibers) were shown in Figure 4. From the response surface plot depicted in Figure 4A,B, lower flow rate resulted in a higher enzymatic activity of nanofibers. It could also be found that the smaller the diameter of the nanofiber, the higher the activity of PAL (Figure 4C–F). The results of this experiment were comparable with the study reported by Chen et al. 2009 [36], indicating that the more surface area of nanofibers, more enzymes can be immobilized on the surface of the nanofibers, thereby higher enzyme activity can be monitored.

### 2.4. Surface Morphology

**Figure 5:**
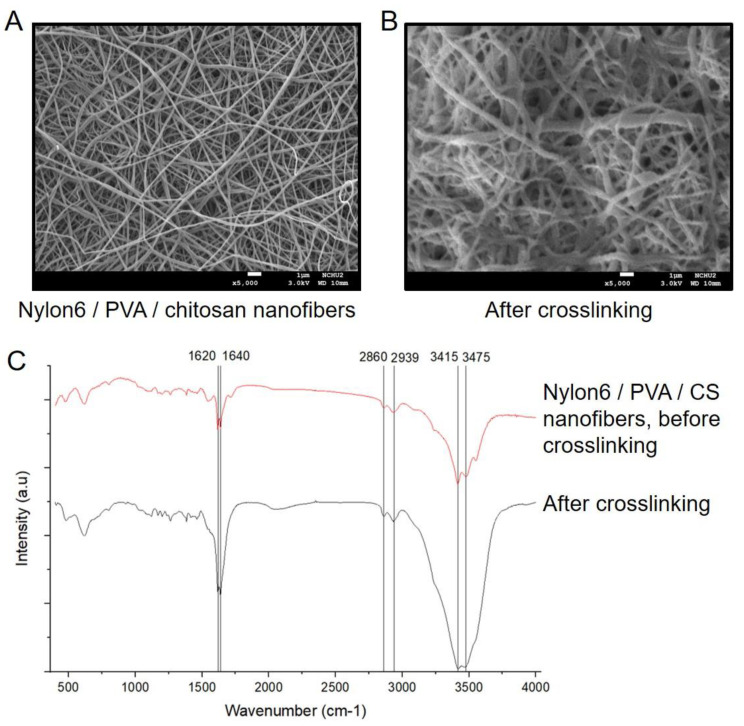
Surface morphology of BoPAL immobilized electrospun nanofibers. Nylon 6/PVA/CS nanofiber (**A**) and crosslinked BoPAL2 nanofiber (**B**) were monitored by thermal field scanning electron microscope. (**C**), Nylon 6/PVA/CS nanofiber and crosslinked BoPAL2 nanofiber were analyzed by Fourier transform infrared spectroscopy.

The prepared PAL/PVA/nylon 6/CS nanofibers were characterized using FE-SEM and FT-IR. In Figure 5A,B, after immobilization, the nanofibers became thicker and more tightly entangled. Since both PAL proteins and crosslinking agent were loaded on the nanofibers, increasing the diameter of nanofibers. In addition, all nanofibers shown in Figure 5C had specific peaks at 2860 and 2939 cm^−1^, which were the characteristic peaks of the CH_2_ tensile vibration of nylon 6 [37]. The bands of 3415 and 3475 cm^−1^ were produced by the superposition of the N−H bond tensile vibrations of chitosan and PAL [38], respectively. The increased intensity of the characteristic peaks at 1620 and 1640 cm^−1^ were attributed to amide group and −N–H stretching vibrations [34]. These phenomena could be confirmed that eBoPAL2 was successfully immobilized on PVA/nylon 6/CS nanofibers through dextran polyaldehyde crosslinking agent.

### 2.5. Temperature and pH Stability of Free and Immobilized BoPAL Proteins

**Figure 6:**
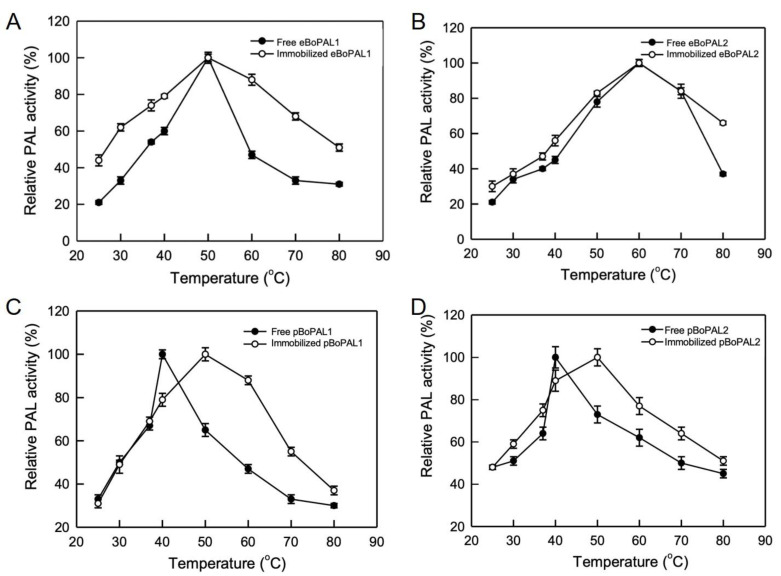
The optimum temperature analysis of free and immobilized BoPAL proteins. (**A**), eBoPAL1; (**B**), eBoPAL2; (**C**), pBoPAL1; (**D**), pBoPAL2. PAL activity of free or immobilized BoPAL protein was performed in standard assay condition within varied temperatures from 25 to 80 °C. The maximum PAL activity was normalized as 100%. All experiments were performed triplicate and expressed as average ± S.D. (error bars).

Optimum reaction temperature of eBoPAL1/2 and pBoPAL1/2 were examined at a range of different temperatures from 25 to 80 °C (Figure 6). The optimum temperatures of free eBoPAL1 and eBoPAL2 were 50 and 60 °C (Figure 6A,B), respectively. The optimum temperatures of free pBoPAL1/2 were both 40 °C (Figure 6C,D), which the optimum temperatures were lower than that of eBoPAL1/2. Immobilized eBoPAL1 showed broader temperature stability than that of free eBoPAL1 (Figure 6A). After protein immobilization, the optimum temperatures of pBoPAL1/2 raised to 50 °C, indicating that compacted/immobilized enzymes can stabilize its protein structures at higher temperature.

**Figure 7:**
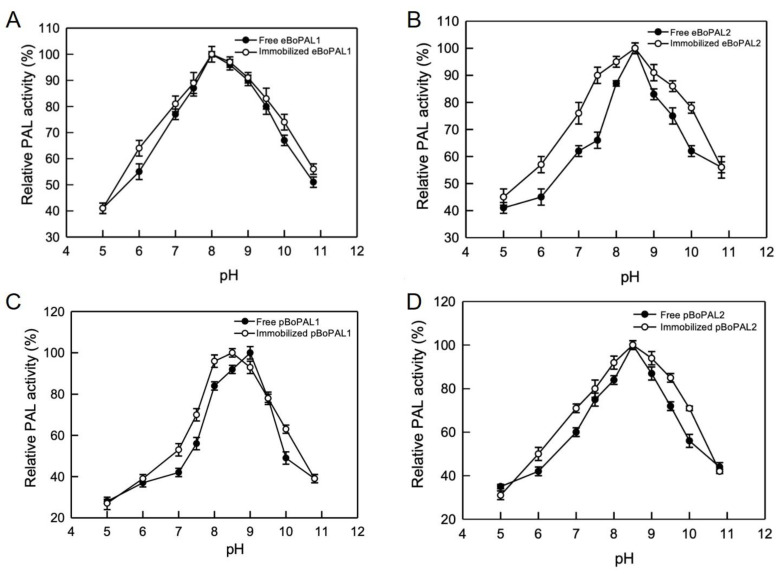
The optimum pH analysis of free and immobilized BoPAL proteins. (**A**), eBoPAL1; (**B**), eBoPAL2; (**C**), pBoPAL1; (**D**), pBoPAL2. PAL activity of free or immobilized BoPAL protein was measured in standard assay condition within varied pH from 5 to 11. The maximum PAL activity was normalized as 100%. All experiments were performed triplicate and expressed as average ± S.D. (error bars).

The optimum pH working conditions for eBoPAL1/2 and pBoPAL1/2 were assessed at the pH from 5 to 11. In Figure 7, the optimum pH of free eBoPAL1 and pBoPAL1 were pH 8.0 and pH 9.0 (Figure 7A,C), respectively, while the optimum pH of free eBoPAL2 and pBoPAL2 was at pH 8.5 (Figure 7B,D). After protein immobilization, PAL activity of all immobilized proteins slightly increased in a wide pH range. The optimum pH of immobilized pBoPAL1 compared to free enzyme was changed from pH 9.0 to pH 8.5 (Figure 7C). It might be caused by the ionization changes of acidic and amino acid side chains in the surrounding environment of the active site of PAL [39,40].

### 2.6. Kinetic Parameters of Free and Immobilized BoPAL Proteins

The *K*_m_ value is an indicator of the affinity between enzyme and substrate; therefore, the larger the *K*_m_ value indicates the lower the affinity. The *k*_cat_ is the turnover number of substrate mole per mole of enzyme per second that can be catalyzed by the enzyme. Thus, the *k*_cat_/*K*_m_ shows the catalytic efficiency and specificity of the enzyme. The *k*_cat_/*K*_m_ value of eBoPAL2 was 1.23 × 10^−2^ s^−1^ μM^−1^ which exhibited better catalytic property than that of other BoPAL proteins (Table 1). In free enzymes, there was no significant difference between BoPAL proteins expressed in *E. coli* and *P. pastoris*. However, the *K*_m_ value of immobilized BoPAL proteins were slightly higher than that of free BoPAL proteins, which might be due to the cross-linking effect that limits the permeability of the matrix [39,40]. In addition, the high porosity of PAL/nylon 6/CS nanofibers allowed the matrix to readily access the active site of PAL. Thus, higher *k*_cat_ values were measured on the immobilized PAL. Taken together, similar *k*_cat_/*K*_m_ values were measured on both free and immobilized BoPALs, suggesting that BoPAL proteins would not greatly reduce catalytic efficiency and specificity due to the cross-linking effect.

### 2.7. Recyclability, Storage Stability, and Denaturant Tolerance of Free and immobiLized BoPAL Proteins

**Figure 8:**
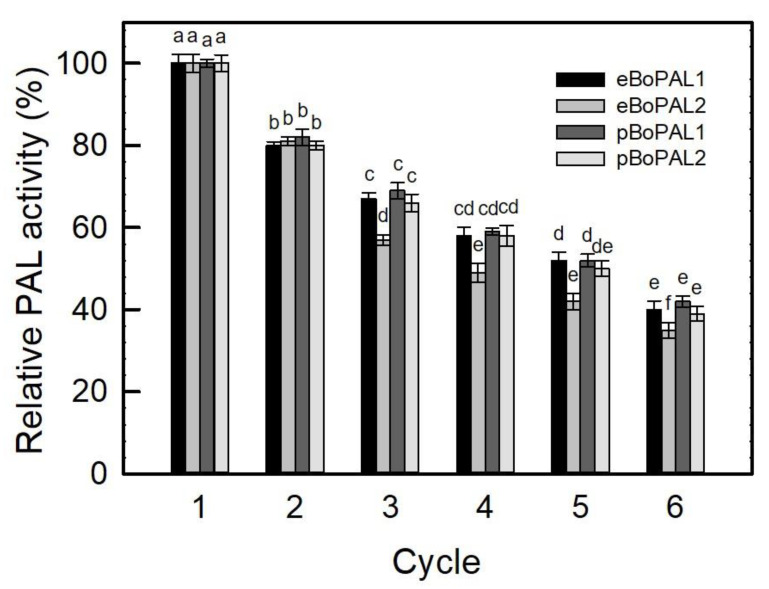
Recyclability of immobilized BoPAL on electrospun nanofibers. PAL activities of immobilized BoPAL proteins were measured in standard assay condition for 30 min followed by transferring to another assay mixture for totally 6 times. All experiments were performed triplicate and expressed as average ± S.D. (error bars). Data were analyzed by one-way ANOVA and different letters were referred to significantly different (*p* < 0.05).

The reusability of immobilized enzymes was a key factor for industrial applications. As shown in Figure 8, after six consecutive cycles of PAL activity determination, there were still 35–42% residual activities on immobilized BoPAL proteins. As a result, PAL/nylon 6/PVA/CS nanofiber was confirmed to possess a certain degree of reusability.

**Figure 9:**
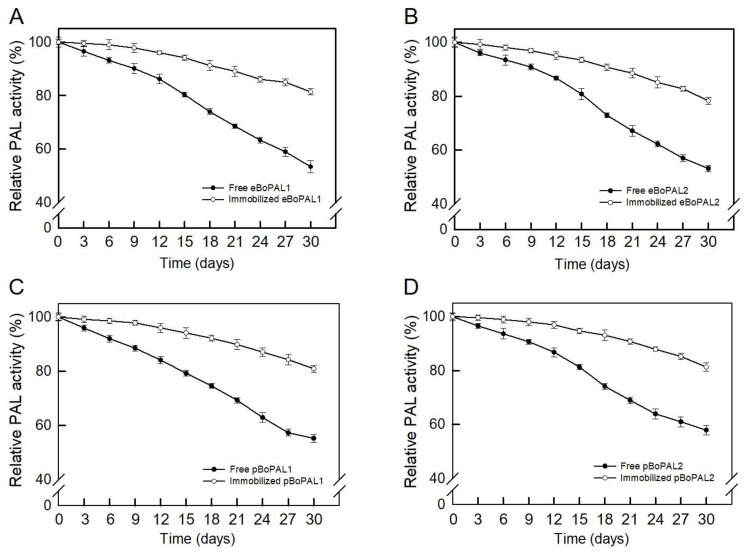
The storage stability of free and immobilized BoPAL proteins. (**A**), eBoPAL1; (**B**), eBoPAL2; (**C**), pBoPAL1; (**D**), pBoPAL2. Samples were stored in 4 °C refrigerator up to 30 days. PAL activity of free or immobilized BoPAL protein was measured in standard assay condition every 3 days. The maximum PAL activity was normalized as 100%. All experiments were performed triplicate and expressed as average ± S.D. (error bars).

To examine storage stability, free and immobilized BoPAL proteins were stored at 4 °C followed by determining PAL activity from 3 days to 30 days (Figure 9). The residual activities of free BoPAL proteins were found to between 48 and 55%, whereas immobilized BoPAL proteins retained 75–83% residual activities (Figure 9A–D). The improved storage stability of immobilized PAL might be due to cross-linking onto PAL/PVA/nylon 6/CS nanofibers which prevents possible distortion effects on the active sites [34].

**Figure 10:**
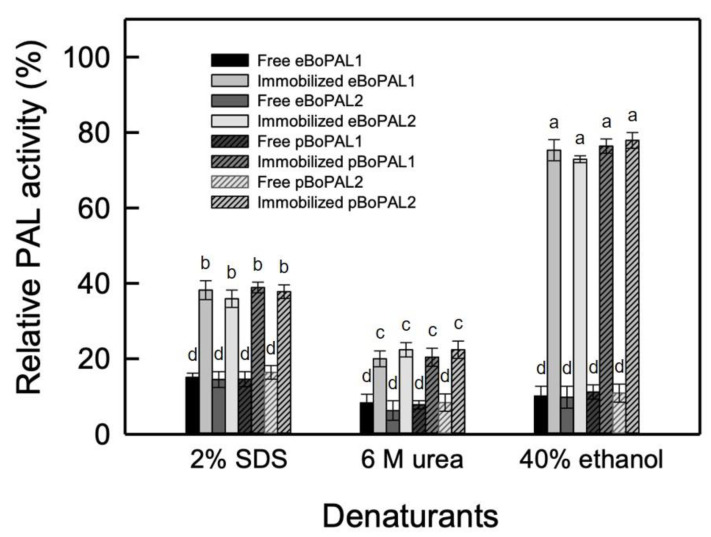
Denaturant tolerance of free and immobilized BoPAL on electrospun nanofibers. PAL activity of free or immobilized BoPAL protein was measured in standard assay condition in the presence of 2% SDS, 6 M urea, or 40% ethanol. All experiments were performed triplicate and expressed as average ± S.D. (error bars). Data were analyzed by one-way ANOVA and different letters were referred to significantly different (*p* < 0.05).

In terms of denaturant tolerance, as the result in Figure 10, the residual activities of free and immobilized PAL were about 15% and 38% in the part of 6 M urea treatment, 8% and 21% in the part of 2% SDS treatment, and 10% and 75% in the part of 40% ethanol treatment, respectively, indicating that PAL/nylon 6/PVA/CS nanofibers are relatively stable to chemical reagents [39,40].

## 3. Discussion

Electrospun nanofibers are characterized by their enhancement for enzyme immobilization through their high surface area and porosity where their functional surface can hold an excessive number of active sites for immobilization [19,22,28,36]. These characteristics can greatly increase the catalytic capacity of the immobilized enzymes [41,42]. BoPAL proteins were immobilized on electrospun nanofibers by using bridging polysaccharide, dextran polyaldehyde, as a macromolecular cross-linker for producing *trans*-cinnamic acid. From the enzyme kinetics, the biochemical properties of recombinant proteins expressed in *E. coli* and *P. pastoris* were comparable as reported before [29,31]. Furthermore, the optimum combinations of electrospun parameters (flow rate 0.10 mL/h, voltage 13.8 kV, and distance 13 cm) were obtained to fabricate nanofiber membranes. Observed by the response surface model, the smaller the fiber diameters was correlated to the higher the PAL activity in the enzyme immobilization.

In 2017, Cui et al., published two important papers, using a simple and low cost method to crosslink *Rhodotorula glutinis* PAL (RgPAL) protein with calcium carbonate (CaCO_3_) for generating enzyme aggregate [39,40]. Compared to free enzyme, RgPAL/CaCO_3_ microsphere exhibited better thermostability, tolerance against chemical denaturants, and mechanical stability. The *K*_m_ and V_max_ values of immobilized RgPAL/CaCO_3_ microsphere were increased and reduced in comparison of free enzyme [39,40]. In this study, we reported alternative enzyme immobilization method and the resulting immobilized BoPAL proteins displayed better thermostability, catalytic activity, recyclability, denaturant tolerance and storage stability than that of free BoPAL proteins. Most importantly, catalytic parameters obtained from free and immobilized BoPALs were comparable, suggesting that dextran polyaldehyde is extremely compatible for immobilizing BoPAL protein on electrospun nanofibers. Compared to traditional chemical crosslinking agent [22,34], this immobilization technique involves biocompatible natural material, it has considerable advantages from environmental concern and increase the usability of the BoPAL proteins.

For now, four BoPAL enzymes were discovered in *Bambusa oldhamii*. The molecular characterization of BoPAL3 protein is not published yet. Among the four BoPAL enzymes, BoPAL4 showed board substrate specificity with L-phenylalanine and L-tyrosine [31]. BoPAL4 can also utilize L-3,4-dihydroxy phenylalanine (L-DOPA) as a substrate to yield caffeic acid (Hsieh, unpublished data). Substrate specificity will be compared in free and immobilized BoPAL4 for a future study.

## 4. Materials and Methods

### 4.1. Reagents

L-phenylalanine, *trans*-cinnamic acid, isopropyl β-D-thiogalactopyranoside (IPTG), Coomassie Brilliant Blue R-250, Coomassie Brilliant Blue G-250, nylon 6, polyvinyl alcohol (PVA, MW 85 kDa–124 kDa), dextran (MW 200 kDa) and chitosan (low molecular weight) were purchased from MilliporeSigma, Burlington, MA, USA. Zeocin was purchased from Invitrogen, Waltham, MA, USA. Protein assay dye reagent concentrate [43], precise plus protein dual colors standards and general chemical reagents for protein electrophoresis were obtained from Bio-Rad, Hercules, CA, USA. All chemical reagents were American Chemistry Society (ACS) or higher grade.

### 4.2. Expressions of BoPAL1 and BoPAL2 Proteins in Escherichia coli and Pichia pastoris

*E. coli* TOP10 bearing with pTrcHis-BoPAL1 or pTrcHis-BoPAL2 plasmid (Table S1) was inoculated in 250 mL of LB medium (0.5% yeast extract, 1% tryptone, 1% NaCl) containing 100 μg/mL of ampicillin and grown at 37 °C on a shaker at 200 rpm overnight. IPTG was added to a final concentration of 1 mM for protein induction. When an OD_600_ of 0.6–1.0 was reached, the cells were further incubated at 30 °C with vigorous shaking for 6 h [29,30,31,33].

*P. pastoris* X-33 inserted with pPICZA-BoPAL1 or pPICZA-BoPAL2 plasmid was selected in YPD-Zeocin medium (1% yeast extract, 2% peptone, 2% dextrose, 100 μg/mL Zeocin) and colonies were transferred onto MD (minimal dextrose) and MM (minimal methanol) plates to further examine their phenotypes in methanol utilization. One colony with Mut^+^ phenotype (methanol utilization plus) was inoculated in 50 mL of BMGY (buffered glycerol-complex medium) and incubated at 30 °C overnight. Cells were precipitated by centrifugation at 1500× *g* for 10 min and then 250 mL BMMY (buffered methanol-complex medium) was added for protein expression at 30 °C overnight. Methanol was continuously added every 24 h to give a final concentration of 0.5% and cells were harvested after 48 h induction [29,31].

### 4.3. Purification of Recombinant BoPAL1 and BoPAL2 Proteins

Recombinant proteins were purified on a Ni-NTA resin (Cyrusbioscience, Taiwan), which was charged with nickel (Ni^2+^) ions. *E. coli* and *P. pastoris* cells were centrifuged at 6000× *g* for 20 min and resuspend in 20 mL 1× lysis buffer (50 mM NaH_2_PO_4_, pH 7.5, 10 mM imidazole, 100 mM NaCl and proper amount of protease inhibitors). *E. coli* and *P. pastoris* cells were mechanically disrupted by sonication using Ultrasonic Processors Sonicator 3000 (Misonix, Farmingdale, NY, USA) and by glass beads (0.5 mm diameter) using Mini-Bead Beater-16 (BioSpec Products, Bartlesville, OK, USA) [44], respectively. The lysate was clarified by centrifugation at 10,000× *g* for 10 min. Then, the supernatant was loaded into a Ni-NTA column and eluted stepwise with buffers containing 50, 125, 250 or 500 mM imidazole. The recombinant proteins extracted from *E. coli* and *P. pastoris* were designated as eBoPAL1/2 and pBoPAL1/2, respectively.

### 4.4. Preparation of Nanofibers by Electrospinning Method

The 4% (*w*/*v*) PVA, 8% (*w*/*v*) nylon 6, and 1% (*w*/*v*) chitosan solution in formic acid were prepared in a water bath set at 60 °C with magnetic agitation for 1 h. After cooling to room temperature, the prepared mixtures were filled in a 5 mL Terumo syringe (21 G, 13 mm inside diameter) and delivered to the spinneret with the typical flow rate maintained at 0.006 mL/min controlled by pump (New Era Pump Systems, Model NE-300, Farmingdale, NY, USA). The spinneret was connected to a high-voltage power supply (Falco, Model FES-COS, New Taipei City, Taiwan) with the applied voltage set at 13.8 kV. The distance between collector plate and the tip of needle was 13 cm. Then, electrospun PVA/nylon 6/CS nanofibers were collected on the baking paper-covered collector plate.

### 4.5. Crosslinking

Dextran polyaldehyde crosslinker was prepared according to Sojitra et al., 2017 [34]. Dextran (1.65 g) and sodium metaperiodate (3.85 g) were added in 50 m1 sodium phosphate buffer (50 mM, pH 6.0). After oxidation for 2 h in the dark condition, 0.3 mL ethylene glycol was added to the reaction mixture to stop the oxidation. Oxidized dextran polyaldehyde solution was dialyzed against sodium phosphate buffer overnight.

Then, PAL protein was immobilized onto nanofiber membrane. First, 3 mg nanofiber membrane was mixed with enzyme solution in 50 mM Tris-HCl buffer, pH 8.5. The reaction mixture was kept for shaking at 150 rpm for 10 min. To determine the optimum immobilization conditions, the following parameters were analyzed: dextran polyaldehyde crosslinker concentration (from 1 to 5%), and crosslinking time (from 3 to 24 h). Finally, the PAL/PVA/nylon 6/CS nanofiber was washed with deionized water and assayed for residual activity using phenylalanine as the substrate under standard PAL activity assay condition.

### 4.6. Central Composite Design (CCD) Response Surface Methodology (RSM)

In this study, CCD-RSM was used to comprehend the optimum combination of parameters in preparation of PAL/PVA/nylon 6/CS nanofibers with the highest enzyme activity. Additionally, the enzyme activity of the produced nanofibers along with their relationship with diameter was studied [36]. Preliminary screening study was ensured that each of the testing parameters was within an appropriate range for electrospinning. The main parameters in electrospinning include the flow rate (X_1_), applied voltage (X_2_), and distance between the spinneret and collector (X_3_). In the part of parameter optimization, eBoPAL2 was used for enzyme immobilization. Then, these parameters were studied at five different levels coded as −α, −1, 0, +1, and +α, which the number of α is (2^3^)^1/4^ = 1.68. The numbers for each parameter, including the central point, corner point and axial point, were set within the particular range by Design-Expert 12.0 (https://www.statease.com/software/design-expert/, accessed on 25 March 2021). PAL activity (Y_1_) and diameter (Y_2_) of nanofibers were selected as response of the system.

Response was fitted to the factors by multiple regressions. The quality of fit of the model was evaluated by the coefficients of determination (R2) and the analysis of variances (ANOVA). The quadratic response surface model was fitted to the following Equation (3):

(3) Y = *β*_0_ + *β*_1_X_1_ + *β*_2_X_2_ + *β*_3_X_3_ + *β*_12_X_1_X_2_ + *β*_13_X_1_X_3_ + *β*_23_X_2_X_3_ + *β*_11_X_1_^2^ + *β*_22_X_2_^2^ + *β*_33_X_3_^2^

In Equation (3), *β*_0_ is the interception coefficient, *β*_1_, *β*_2_, *β*_3_, *β*_12_, *β*_13_, *β*_23_, *β*_11_, *β*_22_, *β*_33_ are the estimated coefficients of the equation.

### 4.7. Membrane Surface morphOlogy Characterization Using Scanning Electron Microscope (SEM) and Fourier Transform Infrared Spectroscopy (FT-IR)

The morphology of the electrospun nanofibers were analyzed by thermal field emission scanning electron microscope (FE-SEM, JEOL JSM-7800F, Akishima, Tokyo, Japan). The diameter of the electrospun nanofiber was determined by ImageJ software (NIH, Bethesda, Maryland, USA). The types of functional groups in the nanofibers were measured by Fourier transform infrared spectroscopy (FTIR) using Spectrum Two^TM^ FTIR Spectrometer (PerkinElmer, Waltham, Massachusetts, USA).

### 4.8. PAL Activity Assay

Protein dye-binding assay [41] was utilized to estimate protein concentrations using bovine serum albumin as reference. PAL enzymatic activity was analyzed by measuring the yield of *trans*-cinnamic acid as the absorbance varied at 290 nm [30]. The reaction mixture contained 50 mM Tris-HCl, pH 8.5, 12.1 mM L-phenylalanine and 50 μL of purified BoPAL enzyme solution in a total volume of 1.0 mL. The reaction mixture was then incubated at 37 °C for 30 min and terminated by adding 100 μL of 6N HCl [33]. D-phenylalanine was used in the parallel assay as a negative control. PAL activity unit (U) was defined as micromoles of *trans*-cinnamic acid formed per minute.

### 4.9. PAL Enzyme Kinetic

To determine the optimum reaction temperature, PAL assays were performed at controlled standard reaction mixture within varied temperatures between 25 and 70 °C. To determine the optimum reaction pH, PAL assays were performed at 37 °C for 30 min using a series of universal buffers (40 mM acetic acid, 40 mM boric acid, and 40 mM phosphoric acid) with varied pH from 5 to 11. To determine the kinetic parameters for PAL, a range of L-phenylalanine were used between 0.12 and 12.1 mM. A substrate saturation curve was obtained after a 10 min incubation [33,45]. The *K*_m_ and *k*_cat_ values were calculated according to Michaelis-Menten equation [46] and double reciprocal plot [47].

### 4.10. Biochemical Properties Analysis

Sodium dodecyl sulfate polyacrylamide gel electrophoresis (SDS-PAGE) was performed according to the methods published before [48,49]. Following electrophoresis, proteins on a gel were stained by Coomassie Brilliant Blue R-250 (CBR) and de-stained by 20% methanol. Gel photo was captured by a Gel Doc XR+ Imaging System (Bio-Rad, Hercules, CA, USA).

### 4.11. Recyclability and Denaturant Treatment

Reusability of immobilized PAL was examined under optimum condition of pH and temperature. The reaction was carried out for 30 min using phenylalanine as a substrate in batch mode. After each cycle, immobilized PAL was re-suspended in fresh substrate solution to determine its residua activity. This process was performed for six consecutive cycles.

PAL activities were compared between free and immobilized PAL in the presence or absence of denaturant. Assays were performed at controlled standard reaction mixture with or without 6 M urea, 2% SDS, or 40% ethanol.

### 4.12. Data Analysis and Statistic

All experiments were performed with triplicate and expressed as mean ± standard deviation (SD, error bars). Graphs and statistical analysis, one-way analysis of variances (ANOVA), were obtained by SigmaPlot software. Different letters were referred to significantly different (*p* < 0.05).

## 5. Conclusions

In this study, the electrospinning parameters such as flow rate, applied voltage, and distance between spinneret and collector were obtained using central composite design-response surface methodology (CCD-RSM) on BoPAL protein. Immobilized enzyme showed better thermostability, catalytic activity, recyclability, tolerance to denaturants and storage stability. Catalytic parameters of free and immobilized BoPAL proteins were corresponding.

## Figures and Tables

**Table 1:** Comparison of the kinetic properties of the free and immobilized BoPAL proteins.

Enzyme	*k*_cat_ (s^−1^)		*K*_m_ (µM)		*k*_cat_/*K*_m_ (s^−1^ µM^−1^)	
	Free	Immobilized	Free	Immobilized	Free	Immobilized
eBoPAL1	1.01	1.21	518	534	1.91 × 10^−3^	2.2 × 10^−3^
eBoPAL2	4.02	3.99	329	379	1.23 × 10^−2^	1.05 × 10^−2^
pBoPAL1	1.03	1.25	376	453	2.72 × 10^−3^	3.32 × 10^−3^
pBoPAL2	3.81	4.01	294	364	1.29 × 10^−2^	1.1 × 10^−2^

## Data Availability

Data is contained within the article or supplementary material.

## Supplementary Materials

The following are available online at https://www.mdpi.com/article/10.3390/ijms222011184/s1.

## Sample Availability

Not available.

## Abbreviations

ANOVA	analysis of variances
BMGY	buffered glycerol-complex medium
BMMY	buffered methanol-complex medium
CBR	Coomassie Brilliant Blue R-25
CCD	central composite design
CS	chitosan
FT-IR	Fourier transform infrared spectroscopy
IPTG	β-D-thiogalactopyranoside
MD	minimal dextrose
MM	minimal methanol
PAL	phenylalanine ammonia-lyase
PVA	polyvinyl alcohol
RSM	response surface methodology
SDS-PAGE	sodium dodecyl sulfate polyacrylamide gel electrophoresis
TFE-SEM	thermal field emission scanning electron microscope
