# Supplementary material for: Production of Trans-Cinnamic Acid by Immobilization of the Bambusa oldhamii BoPAL1 and BoPAL2 Phenylalanine Ammonia-Lyases on Electrospun Nanofibers
Source: Int J Mol Sci. 2021 Oct 17;22(20):11184. doi: 10.3390/ijms222011184 (PMC8541413; doi:10.3390/ijms222011184)
Supplement: Supplementary file 1 [file ijms-22-11184-s001.zip › ijms-1397873-supplementary.pdf]

Table S1. Plasmids used for bamboo BoPAL1 and BoPAL2 expressions in *Escherichia coli* and *Pichia pastoris*

Plasmids	Relevant characteristics	Source/Ref.
pTrcHisA	*E. coli* expression vector with N-terminal His_6-tag fusion	Invitrogen
pTrcHisA-BoPAL1	*BoPAL1* coding sequence inserted into pTrcHisA for eBoPAL1 expression	31
pTrcHisA-BoPAL2	*BoPAL2* coding sequence inserted into pTrcHisA for eBoPAL2 expression	29
pPICZA	*P. pastoris* expression vector with C-terminal His_6-tag fusion	Invitrogen
pPICZA-BoPAL1	*BoPAL1* coding sequence inserted into pPICZA for pBoPAL1 expression	31
pPICZA-BoPAL2	*BoPAL2* coding sequence inserted into pPICZA for pBoPAL2 expression	29

Table S2. Ranges of experimental parameters of five-level-three-factor central composite design

	Levels				
Factors	-1.68	-1	0	1	1.68
X_1: Flow rate (ml/h)	0.08	0.09	0.105	0.12	0.13
X_2: Voltage (kV)	11	12	13.5	15	16
X_3: Distance (cm)	8.6	10	12	14	15.3

Table S3. Central composite design and experimental data of immobilized PAL activity and nanofiber diameter

Run	X_1: Flow rate (ml/min)	X_2: Voltage (kV)	X_3: Distance (cm)	Y_1: PAL activity (μU/mg NF)[1]	Y_2: Diameter (nm)
1	0	0	0	250	127
2	0	0	0	240	122
3	0	0	0	235	122
4	0	0	0	239	113
5	1	1	-1	181	155
6	0	0	-1.68	192	138
7	-1	1	-1	203	136
8	1.68	0	0	184	171
9	-1.68	0	0	215	131
10	0	-1.68	0	191	137
11	-1	1	1	234	124
12	0	0	0	241	125
13	-1	-1	1	235	123
14	1	-1	-1	199	175
15	1	-1	1	192	129
16	0	0	0	238	112
17	0	1.68	0	225	149
18	1	1	1	192	114
19	0	0	1.68	226	115
20	-1	-1	-1	170	152

[1] PAL activity unit (U) is defined as 1 mole *trans*-cinnamic acid formation per minute. NF, <u>n</u>ano<u>f</u>iber.
